# Protocol to identify and sort antigen-specific B cells from *Plasmodium*-infected mice

**DOI:** 10.1016/j.xpro.2025.104129

**Published:** 2025-10-07

**Authors:** Carolina Calôba, Allen M. Minns, Scott E. Lindner, Rahul Vijay

**Affiliations:** 1 Discipline of Microbiology and Immunology, Rosalind Franklin University of Medicine and Science, North Chicago, IL, USA; 2 School of Graduate and Postdoctoral Studies, Rosalind Franklin University of Medicine and Science, North Chicago, IL, USA; 3 Center for Cancer Cell Biology, Immunology and Infection, Rosalind Franklin University of Medicine and Science, North Chicago, IL, USA; 4 Department of Biochemistry and Molecular Biology, Huck Center for Malaria Research, Pennsylvania State University, University Park, PA, USA; 5 Foundational Science and Humanities, Rosalind Franklin University of Medicine and Science, North Chicago, IL, USA

**Keywords:** Immunology, Microbiology, Model Organisms

## Abstract

Here, we detail a protocol to identify an antigen-specific B cell population using SpyCage reagents tailored to bind MSP_1-19_ antigen in a rodent malaria model. We describe steps for the preparation of splenic single-cell suspension, B cell enrichment, and staining to facilitate sorting of live cells for downstream applications such as single-cell RNA, V(D)J, and assay for transposase-accessible chromatin (ATAC) sequencing.

For complete details on the use and execution of this protocol, please refer to Calôba et al.^1^

## Highlights

• Steps for preparing single-cell suspension and antigen-specific B cell enrichment
• Instructions for optimizing staining of compensation/reference control beads
• Guide to sort-purify antigen-specific B cells for downstream workflow

## Abstract (editor highlights)

Publisher’s note: Undertaking any experimental protocol requires adherence to local institutional guidelines for laboratory safety and ethics.

## Graphical abstract

**Figure:**
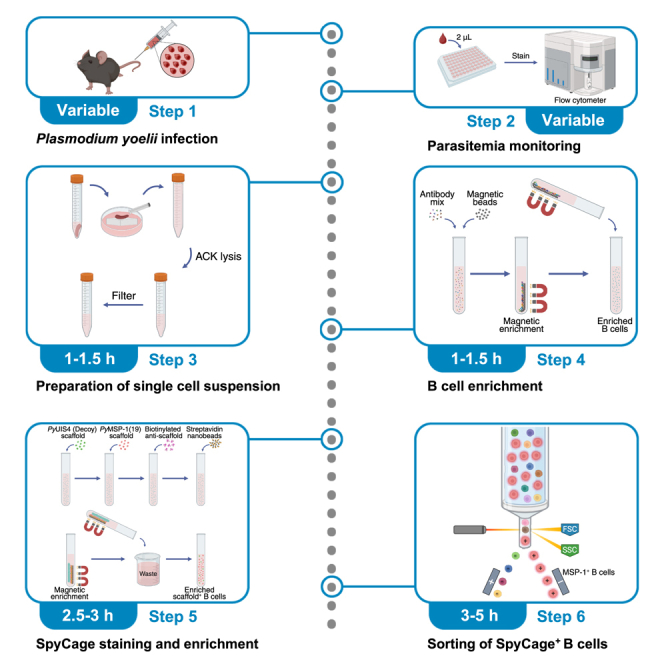

## Before you begin

B cells play a critical role in protection against malaria by secreting antibodies specific to a wide range of *Plasmodium* antigens.^2^^,^^3^^,^^4^^,^^5^^,^^6^^,^^7^^,^^8^ The protocol below describes the specific steps for infection of mice with *Plasmodium yoelii-*17XNL Clone 1.1 *(Py)*, spleen harvest, preparation of splenic single cell suspension, staining of MSP_1-19_-specific B cells, as well as sorting live cells. Stained cells can be either used for phenotypic characterization using flow cytometry or for transcriptomic and epigenetic analysis using single-cell sequencing techniques. While we used this methodology to characterize memory B cells from *Plasmodium*-infected mice, this approach can be broadly applied to identify non-*Plasmodium* antigens in mice, when the right combination of “SpyCage” reagents is used.

1. Prepare required buffers as described below.
2. If needed, perform a trial run of the experiment to ascertain the minimum quantity of antibodies and enrichment kits necessary, as the size of the spleen and, therefore, the number of cells will vary depending on the time point of harvest post infection.

### Institutional permissions

C57BL/6 mice were purchased from The Jackson Laboratory, and *Plasmodium yoelii* clone 17xNL (*Py*) was obtained from the Malaria Research and Reference Reagent Resource center (MR4; BEI Resources, American Type Culture Collection). All experiments and procedures were approved by the Rosalind Franklin University of Medicine and Science Institutional Animal Care and Use Committee. Researchers who plan to execute this protocol must obtain approval from their institutional committees.

### Innovation statement

This protocol is innovative in that it employs highly sensitive reagents to detect *bona fide* parasite-specific germinal center (GC) B cells and memory B cells (MBCs). These fluorescent protein–labeled SpyCage reagents are significantly brighter than conventional B cell tetramers, enabling confident identification of antigen-specific B cell populations, even when using a semi–high-throughput flow cytometry panel. Combined with a carefully optimized workflow-refined through multiple rounds of trial and error, this protocol enables high-resolution analysis of the phenotypic and molecular characteristics of antigen-specific MBCs during *Plasmodium* infection.

### *Plasmodium yoelii* infection and assessment

**Timing: variable**

This step describes how to infect mice with *Py* and measure parasitemia by flow cytometry during the course of infection.^9^ Perform the following procedures in an ABSL2 facility.

3. *Py* preparation and infection.
  a. Thaw *Py* stock stored in liquid nitrogen in a water bath at 37°C.
  b. Dilute *Py* stock with sterile PBS to achieve a concentration of 5x10^6^ parasitized red blood cells (RBC) per mL.
  c. Inject 200 μL (1x10^6^ parasitized red blood cells) of the diluted *Py* stock into each mouse either intravenously (i.v.) or retro-orbitally (r.o.).

***Note:*** Place the mouse under an infrared lamp (25–30 cm away) briefly (1–3 min) to facilitate vasodilation of the peripheral (tail) veins. Transfer the mouse into a mouse restrainer and sterilize the injection site (tail) using gauze soaked in 70% v/v ethanol before injection with a sterile 26-gauge syringe. Upon successful completion of the injection, slowly withdraw the needle and apply slight pressure to the injection site to prevent bleeding. Alternatively, perform r.o. injections in mice under controlled isoflurane anesthesia.

**Figure 1:**
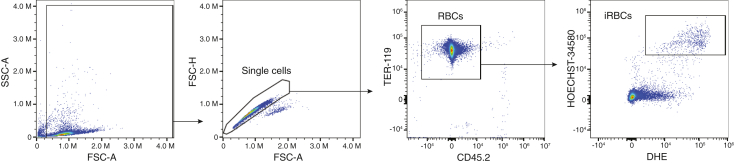
Assessment of parasitemia using flow cytometry Gating strategy used to obtain the fraction of infected red blood cells (iRBC).
k. Repeat parasitemia measurements every other day to monitor the infection until the mice clear the infection or until the desired day of harvest.

4. Monitor parasitemia (starting on day 5 post infection).

a. Aliquot 100 μL of PBS per well in a 96-well flat-bottom plate. Keep the plate on ice. The number of wells should be equal to the number of infected mice plus one extra well to be used as an unstained control.
b. Place a mouse into a mouse restrainer and snip the tip of the tail carefully using sharp and sterile scissors. Milk the tail gently from the base to the tip and collect blood using a 10 μL pipette set to 2 μL. Transfer the blood to the corresponding well containing 100 μL sterile PBS and mix by pipetting.
c. Add at least 1 μL of blood from one mouse per group to the well for the unstained control.
d. Add 100 μL of cold PBS and centrifuge at 200 *xg* for 5 minutes at 4°C.
e. Discard supernatant and pulse vortex the plate.
f. Prepare the staining mix with the following antibodies and dyes:
  i. Hoechst-34580 (1:1000 dilution, BV570 on Cytek Aurora).
  ii. Dyhydroethidium (DHE) (1:500 dilution, BB630 on Cytek Aurora).
  iii. APC-conjugated anti-Ter119 (1:400).
  iv. FITC-conjugated CD45.2 (1:200).
    **CRITICAL:** Preparation of the staining mix is done in PBS.
g. Add 100 μL of staining mix per well and incubate for 20 minutes at 4°C in the dark.
h. Wash wells using 100 μL of cold PBS and centrifuge at 200 *xg* for 5 minutes at 4°C.
i. Resuspend in 200 μL of cold PBS and acquire in a flow cytometer as soon as possible.
j. Data is analyzed using FlowJo software as illustrated in Figure 1.

## Key resources table

**Table:** 

REAGENT or RESOURCE	SOURCE	IDENTIFIER
**Antibodies**		

Anti-mouse F4/80, PE/Cy7-conjugated (clone BM8) (1:250)	BioLegend	Cat #: 123114; RRID:AB_893478
Anti-mouse CD38, APC-conjugated (clone 90) (1:250)	BioLegend	Cat #: 102712; RRID:AB_312932
Anti-mouse IgD, Spark NIR 685-conjugated (clone 11-26c.2a) (1:500)	BioLegend	Cat #: 405750; RRID:AB_2888693
Anti-mouse CD3, APC/Fire 810-conjugated (clone 17A2) (1:200)	BioLegend	Cat #: 100268; RRID:AB_2876392
Anti-mouse B220, PerCP/Cy5.5-conjugated (clone RA3-6B2) (1:300)	BioLegend	Cat #: 103235; RRID:AB_893354
Anti-human/mouse GL7, eFluor 450-conjugated (clone GL-7) (1:300)	Invitrogen	Cat #: 48-5902-82; RRID:AB_10870775
Anti-mouse CD45.2, FITC-conjugated (clone 104) (1:200)	BioLegend	Cat #: 109806; RRID:AB_313442
Anti-mouse TER-119, APC-conjugated (clone TER-119) (1:400)	BioLegend	Cat #: 116212; RRID:AB_313712
FcBlock (CD16/32) (2 μg/sample)	Prepared in the lab	N/A

**Chemicals, peptides, and recombinant proteins**		

Dihydroethidium	Sigma-Aldrich	Cat #: D70008
Hoechst 34580	Sigma-Aldrich	Cat #: 63493
MSP1-19 SpyCage	Calôba et al.^1^	N/A
*Py*UIS4 SpyCage	Calôba et al.^1^	N/A
HEPES	Corning	Cat #: 25-060-CI
L-glutamine 200 mM	Gibco	Cat #: 25030-081
Penicillin/streptomycin	Gibco	Cat #: 15140-122
Gentamicin	Gibco	Cat #: 15710-064
b-mercaptoethanol	Sigma-Aldrich	Cat #: M3148-100ML
KHCO_3_	Fisher Chemical	Cat #: P235-500
NH_4_Cl	Mallinckrodt Chemicals	Cat #: 3384
EDTA-Na_2_	Sigma-Aldrich	Cat #: ED2SS
Zombie Aqua fixable viability kit	BioLegend	Cat #: 423102

**Critical commercial assays**		

MojoSort mouse pan B cell isolation kit II	BioLegend	Cat #:480088
MojoSort streptavidin nanobeads	BioLegend	Cat #: 480016
Protector RNase inhibitor	Sigma-Aldrich	Cat #: 03335399001 Sigma-Aldrich

**Experimental models: Organisms/strains**		

Model organism: mouse: C57BL/6 (WT), males or females, 6- to 8-weeks old	Jackson Laboratory	Stock: 00664
*Plasmodium yoelli 17XNL* (clone 1.1)	BEI Resources	MRA-593

**Software and algorithms**		

SpectroFlo	Cytek Biosciences	N/A
FlowJo v.10.10.0	BD	https://www.flowjo.com
BioRender	N/A	https://app.biorender.com

**Other**		

RPMI-1640 medium	Sigma-Aldrich	Cat #: R8758-500ML
Fetal bovine serum (FBS)	Gibco	Cat #: 26140-079
PBS	Cytiva	Cat #: SH30028.03

## Materials and equipment

**Table:** SC (Supplementum Complementum)

Reagent	Final concentration	Amount
RPMI-1640 Medium	N/A	200 mL
HEPES (1 M)	0.2 M	100 mL
L-Glutamine 200 mM	40 mM	100 mL
Penicillin/Streptomycin	2000 U/mL / 2000 μg/mL	100 mL
Gentamicin	0.2 mg/mL	10 mL
β-Mercaptoethanol	2 mM	70 μL

• Prepare SC stock to be used when preparing RP-1 medium.
• Filter through 0.22 μM filter.
• Aliquot 12.5 mL into 15 mL tubes for storage.

***Note:*** Store SC at −20°C until use.

**Table:** RP-1 medium

Reagent	Final concentration	Amount
RPMI-1640 Medium	N/A	482.5 mL
SC	1X	12.5 mL (1 aliquot)
Fetal Bovine Serum	1% v/v	5 mL

***Note:*** Store RP-1 at 4°C for not more than a month.

**Table:** ACK Lysis Buffer

Reagent	Final concentration	Amount
ddH_2_O	N/A	1 L
KHCO_3_	0.01 M	1 g
NH_4_Cl	0.15 M	8.29 g
EDTA-Na_2_	0.0001 M	37.2 mg

• Prepare ACK Lysis buffer stock to use for RBC lysis.
• Adjust pH to 7.2–7.4 at room temperature with HCl before increasing the volume to 1 L.
• Filter through 0.22 μM filter.
• Aliquot 50 mL into 50 mL tubes for storage.

***Note:*** Store ACK Lysis buffer at 25°C until use.

## Step-by-step method details

### Preparation of single-cell suspension from infected spleens

**Timing:** 1–1.5 h

This step outlines how spleens are processed into a single cell suspension, followed by red blood cell (RBC) lysis. Please note that these steps are performed on the day post infection that pertains to your experimental question. For our specific application, these steps were conducted on 38 days post infection (dpi).

1. Euthanize mice:
  a. Sacrifice mice using CO_2_ inhalation in a chamber until the animals become unconscious and cease to move.
  b. To confirm euthanasia, perform cervical dislocation.
2. Collect spleens into tubes containing 3 mL of RP-1 on ice.
3. Process the tissues:
  a. Transfer the spleen and RP-1 to a 10 cm petri dish containing a 180 μm wire mesh screen molded into a strainer.
  b. Using a syringe piston, push the spleens through the wire mesh strainer.
  c. Force 4 mL of fresh RP-1 through the wire mesh with the pipette gun set to high speed.
  d. Aspirate back the entire volume (7 mL) and wash the wire mesh again. Repeat this process at least 3 times.
  e. Transfer the cells into a 15 mL tube.
  f. After transferring the cells, repeat steps c to e.

**CRITICAL:** The washes are important to guarantee a high yield of cells.

4. Lyse the RBCs:
  a. Centrifuge cells from step 3f at 300 *xg* for 5 minutes at 4°C.
  b. Dislodge the pellets by vortexing or by sliding the tubes against a tube rack.
  c. Add 1 mL of ACK lysis buffer. Mix it well using a P1000 pipette.
  d. Add an additional 2 mL of ACK lysis buffer and mix well.
    ***Note:*** The volume of ACK lysis buffer depends on the size of the spleen. For instance, for larger spleens from 15 dpi (*Py*) we usually add a total of 4 mL of ACK lysis buffer, while smaller spleens from a memory time point (post 35 dpi) will require 3 mL of ACK lysis buffer for optimal RBC lysis.
  e. Incubate for 2–3 minutes at 25°C.
    **CRITICAL:** Longer incubation times at 25°C may result in loss of immune cells. Incubation on ice may require longer times, which may also result in loss of immune cells.
  f. Add fresh RP-1 to a final volume of 14 mL to neutralize the ACK lysis buffer.
  g. Centrifuge cells at 300 *xg* for 5 minutes at 4°C.
  h. Resuspend in 5 mL of RP-1 and filter the cells through a 70-micron filter mesh into a new 15 mL tube. Keep the tubes on ice.
  i. Take a 10 μL aliquot and count the cells.

### B cell enrichment and live/dead staining

**Timing: 1–1.5 h**

This step describes the steps involved in enriching B cells through negative selection using magnetic beads and performing live/dead stain before staining for antigen-specific B cells using SpyCage reagents. We followed the manufacturer’s protocol as outlined below.

5. Prepare cells for enrichment using MojoSort Pan B cell isolation kit.
  a. Centrifuge the single cell suspension from step 4 h at 300 *xg* for 5 minutes at 4°C.
  b. Discard supernatant and resuspend cells in RP-1 to a final concentration of 1x10^8^ cells/mL and transfer to a 5 mL polystyrene (FACS) tube.
  c. Add 100 μL of antibody mix/mL and mix with a P1000 pipette.
  d. Incubate for 15 minutes on ice.
  e. Add 100 μL of nanobeads/mL and mix with a P1000 pipette.
  f. Incubate for 15 minutes on ice.
  g. Add RP-1 to a final volume of 2.5 mL, mix with a P1000 pipette, and place the cells in the MojoSort magnet for 5 minutes.
  h. Without removing the tube from the magnet, hold the magnet and swiftly pour the liquid into a new 15 mL tube.
    **CRITICAL:** The transferred liquid is the enriched fraction; do not discard.
  i. Remove the tube from the magnet and add an additional 2.5 mL of fresh RP-1.
  j. Resuspend the beads and place the tube in the magnet for 5 minutes.
  k. Without removing the tube from the magnet, pour the liquid into the 15 mL tube (from step 5h) already containing 2.5 mL and discard the beads. Keep on ice.
  l. Take an aliquot of cells to use as the unstained reference control.
  m. Take a 10 μL aliquot and count the cells.
    ***Note:*** Other Pan B cell isolation kits should work besides the MojoSort Pan B cell enrichment kit.
    **CRITICAL:** If using any other B cell enrichment kits, be sure to use negative selection kits.
  n. Add ice-cold PBS to the tube from step 5k to a final volume of 15 mL.
  o. Centrifuge the cells at 300 *xg* for 5 minutes at 4°C.
  p. Resuspend cells in 15 mL of PBS and repeat step 5n.
    **CRITICAL:** Washing steps with PBS before proceeding to the next step (Live/Dead staining) are critical since the presence of serum will result in suboptimal Live/Dead staining.
6. Stain cells with Live/Dead staining.
  a. Resuspend cells in 1 mL of diluted Live/Dead dye.
    ***Note:*** Live/Dead dye (Zombie Aqua Fixable Viability Kit) should be diluted at 1:1000 in PBS.
    **CRITICAL:** Use PBS to dilute live/dead dye. Do not use media containing serum.
  b. Incubate for 20 minutes at 25°C in the dark.
  c. Add fresh RP-1media to a final volume of 15 mL to neutralize Live/Dead stain.

### SpyCage staining and enrichment

**Timing: 2.5–3 h**

B cells are stained with SpyCage reagents along with other surface markers and the SpyCage^+^ cells are enriched through positive selection using magnetic beads.

***Note:*** The SpyCage reagent has been used in our prior studies to nanoparticle vaccine approaches in the context of SARS-CoV-2.^10^ SpyCage consists of the fusion of the SpyCatcher domain to the N-terminus of the engineered I3-01 protein, with two 6xHis tags present for protein purification.^11^^,^^12^ We created two variants of the SpyCage reagents for this study by appending additional sequences to the C-terminus of I3-01, either with mScarlet to create “RedCage” – or with mNeonGreen to create “GreenCage”. In this study, the RedCage was used as the antigen-specific experimental reagent by covalently loading it with 60 copies of the targeted immunodominant *Py* antigen MSP_1-19_, capable of detecting MSP_1-19_ specific B cells. The GreenCage was used as a decoy control and was covalently loaded with 60 copies of the liver-stage antigen *PyUIS4*, which is not expressed in the blood stage. The *Py*MSP_1-19_ (AA1619-1754) and *PyUIS4* (AA80-224) antigens were expressed in *E. coli* as GST-fusion proteins bearing a C-terminal SpyTag to enable covalent bonding to SpyCage.^13^ The SpyCage scaffold is designed to be versatile and to accommodate a wide range of antigens for display. Antigens are produced separately, and loaded onto SpyCage simply by mixing in a compatible buffer (*e.g.* 1x PBS) and temperature (*e.g.* 4^o^C–37^o^C). Unbound antigens were purified away via standard column chromatography and/or dialysis approaches.

7. Stain cells with SpyCage reagent.
  a. Centrifuge cells (from step 6c) at 300 *xg* for 5 minutes at 4°C.
  b. Discard the supernatant and resuspend cells in 1 mL of RP-1 and add 2 μg of FcBlock (CD16/32) and 1.25 μg of the decoy “GreenCage” conjugated to PyUIS4 antigen. Pulse vortex the tubes 5 times gently.
  c. Incubate for 10 minutes at 25^o^C in the dark.
  d. Add 1.25 μg of the RedCage conjugated to *Py*MSP1(19) and gently vortex the tubes.
  e. Incubate for 30 minutes at 4^o^C in the dark.
  f. Add fresh RP-1 media to a final volume of 15 mL.
  g. Centrifuge at 300 *xg* for 5 minutes at 4^o^C.
  h. Discard the supernatant and resuspend cells in 2 mL of RP-1.
  i. Add 1 μg of biotinylated anti-scaffold antibody and gently vortex the tubes.
  j. Incubate for 30 minutes at 4^o^C in the dark.
  k. Add fresh RP-1 media to a final volume of 15 mL.
8. Enrich cells bound to the SpyCage scaffold.
  a. Centrifuge cells at 300 *xg* for 5 minutes at 4^o^C.
  b. Discard the supernatant and resuspend cells at 1x10^8^ cells/mL and add 100 μL of MojoSort streptavidin nanobeads/mL.
  c. Incubate for 15 minutes at 4^o^C in the dark.
  d. Add fresh RP-1 media to a final volume of 15mL.
  e. Centrifuge at 300 *xg* for 5 minutes at 4^o^C.
  f. Discard the supernatant and resuspend cells in 2.5 mL of RP-1 and transfer to a polystyrene (FACS) tube.
  g. Place the tube in the magnet and incubate for 5 minutes.
  h. Without removing the tube from the magnet, hold the magnet and swiftly pour the unlabeled fraction into another FACS tube.
  i. Remove the tube from the magnet and add 1 mL of RP1 into the tube to prevent cells from drying and keep it on ice. This is ‘labeled fraction 1’.
    ***Note:*** The labeled fraction 1 is enriched for SpyCage^+^ cells.
  j. Place the FACS tube from step 8h containing the unlabeled cells back in the magnet and incubate for 5 minutes.
  k. Without removing the tube from the magnet, hold the magnet and swiftly pour and discard the unlabeled fraction.
  l. Remove the tube from the magnet and add 1 mL of RP1 into the tube to prevent cells from drying, and combine with ‘labeled fraction 1’ from step 8i to a final volume of 2 mL.

### Sorting of SpyCage^+^ B cells

**Timing: 3–5 h**

Enriched SpyCage^+^ B cells are stained with other relevant surface markers to sort-purify your population of interest.

9. Stain B cells for sorting.
  a. Centrifuge cells from step 8l at 300 *xg* for 5 minutes at 4^o^C.
  b. Resuspend in 500 μL of antibody cocktail mix. The antibody cocktail will contain the following antibodies resuspended in RP-1:
    i. B220 PerCP/Cy5.5 (1:300).
    ii. IgD Spark NIR (1:800).
    iii. GL7 eF450 (1:300).
    iv. CD38 APC (1:250).
    v. CD3 APC/Fire 810 (1:200).
    vi. F4/80 PE/Cy7 (1:250).
      ***Note:*** One may add more markers depending on the populations of interest.
      **CRITICAL:** Remember not to add any markers tagged with Spark YG593 or FITC or any fluorophores whose emission spectra peaks at YG2 or B2 on a Cytek instrument. These channels have already been used for the two SpyCage scaffolds. If using a different instrument, be sure to identify the respective channels.
      ***Optional:*** If one of the downstream applications involves CITE-seq, CITE-seq antibodies should be added during this step at 1:1000 dilution or as recommended by the manufacturer.
  c. Incubate for 30 minutes at 4^o^C in the dark.
  d. Add 3 mL of RP-1 to wash.
  e. Centrifuge at 300 *xg* for 5 minutes at 4^o^C.
    ***Note:*** If transcriptomic analysis is one of the downstream applications, add Protector RNase Inhibitor to fresh RP-1 media at this point. This media will be used to resuspend the cells for sorting and should contain 1 U/mL of Protector RNase Inhibitor. The media in the collection tubes should contain 0.5 U/mL of Protector RNase Inhibitor. For other applications, fresh RP-1 media will be sufficient.
  f. Discard the supernatant and resuspend cells at 1x10^7^ cells/mL of the appropriate media. Keep on ice.
  g. Add 500 μL of the appropriate media to the collection tubes. Vortex the tubes to coat their walls. Keep on ice.
  h. Proceed to cell sorting.
    ***Note:*** It is ideal to use a sorting nozzle with a pore size greater than 70 μm. In this protocol, we used a 100 μm nozzle. It is also important to gate on CD3^−^F4/80^−^ cells before proceeding with B cell gates.
    **CRITICAL:** While preparing single stained (compensation or reference) controls to be used in the cell sorter, use compensation beads such as UltraComp eBeads (Thermo Fisher) or similar. It is also critical that when beads are prepared with SpyCage reagents, first add biotinylated anti-aldolase antibody and then the respective SpyCage scaffolds. For all other markers in the staining panels, stain as usual.
  i. Proceed to the downstream workflow as desired.

## Expected outcomes

**Figure 2:**
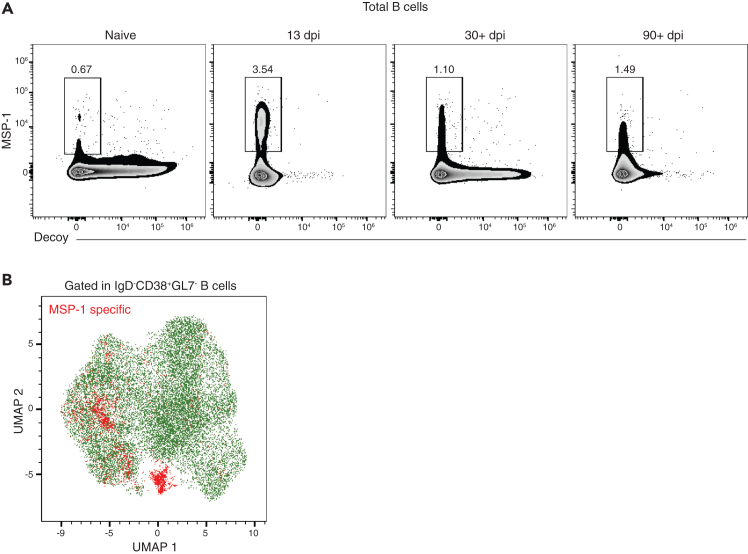
MSP-1 staining (A) Representative plots of total B cells stained using SpyCage reagents on different time points of *Py* infection. (B) UMAP of memory B cells stained with SpyCage reagents and analyzed by flow cytometry on 38 dpi.

This protocol enriches and stains for MSP_1-19_-specific B cells, which can be analyzed by flow cytometry or flow sorted and used for other downstream applications such as sequencing. We have also used this protocol in combination with transcription factor staining. A successful SpyCage staining will show a clear single positive MSP-1 population (Figure 2A). As expected, MSP_1-19_-specific B cells will be higher in frequency during the effector phase of infection and will contract during the memory time point. It is important to note that the frequency of MSP_1-19_-specific B cells is usually low (below 5%) even after the enrichment step. Clustering of MSP_1-19_-specific memory B cells can clearly detect the stained population among the other B cell populations, as shown in Figure 2B. Please refer to Calôba et al. for a more detailed high-dimensional analysis of MSP_1-19_-specific memory B cells.

## Quantification and statistical analysis

FlowJo software (v.10.10.0, BD) was used for analysis of flow cytometry data.

## Limitations

Given the low relative frequency of MSP_1-19_-specific B cells, the success of this protocol critically depends on the efficient isolation of SpyCage-specific B cells using magnetic beads. It is important to note that, even after enrichment, the isolated cells will still contain other B cell subsets. However, SpyCage-specific B cells will be substantially enriched, allowing for their visualization and, if needed, further purification by cell sorting. For downstream applications such as sequencing, samples can be pooled from at least 3 mice to achieve the amount of cells needed.

This protocol has been validated only in mice infected with *Plasmodium yoelii*. Although there may be sequence similarities in the MSP_1-19_ antigen across different *Plasmodium* species,^14^ we have not yet assessed the reliability of this antigen for identifying and isolating antigen-specific B cells in mice infected with other strains.

## Troubleshooting

### Problem 1

Unable to visualize/identify MSP_1-19_ specific B cells.

### Potential solution

The above-mentioned issue may arise due to suboptimal reference/compensation bead staining (step 9). As mentioned elsewhere in the protocol, staining with individual SpyCage reagents is a two-step process. The first step uses a fluorescently labeled scaffold, followed by the use of an antibody that is specific to the scaffold. Thus, when staining beads for reference/compensation control to be used on a flow cytometer, reverse the staining sequence - stain with the anti-aldolase antibody first, followed by the aldolase scaffold. This order is important because the beads will be unable to bind directly to the fluorescently labeled scaffold without being bound to the anti-aldolase antibody, leading to a complete absence of positively stained population.

### Problem 2

Very few MSP_1-19_ specific B cells are detected.

### Potential solution

Suboptimal RBC lysis (step 4). Depending on the time point of infection, one may have to add more ACK lysis buffer (up to 5 ml) and incubate for longer periods (up to 4 minutes) to get effective lysis of RBCs. Please be sure to incubate cells with RBC lysis buffer (ACK lysis buffer) at room temperature.

### Problem 3

Dimmer fluorescence signal from SpyCage reagents.

### Potential solution

Adjusting the autofluorescence extraction threshold prior to unmixing and identifying/sorting cells of interest (in a spectral flow cytometer).

### Problem 4

Lower quality of RNA or cDNA library.

### Potential solution

Ensuring that RNase inhibitor (step 9) added to the cell suspension is not expired or denatured.

### Problem 5

MSP-1^+^ cells do not show a bimodal staining pattern but rather shows a spread.

### Potential solution

This can be minimized by adding recommended amount of Fc block (step 7) as well as ensuring that F4/80 (step 9h) is used a dump gate before proceeding with B cell gates.

## Resource availability

### Lead contact

Requests for further information should be directed to and will be fulfilled by the lead contact, Rahul Vijay, rahul.vijay@rosalindfranklin.edu.

### Technical contact

Requests for further information should be directed to corresponding authors Scott E. Lindner (scott.lindner@psu.edu) and Rahul Vijay (rahul.vijay@rosalindfranklin.edu).

### Materials availability

Please contact Scott E. Lindner (scott.lindner@psu.edu) for inquiries related to SpyCage, PyMSP1(19), and PyUIS4 proteins used in this study.

### Data and code availability

This paper did not generate any new datasets or code.

## Acknowledgments

This work was supported by 1R01GM125907 to S.E.L., the RFUMS startup funds, Schweppe Scholar Award 1R01AI182217 to R.V., and the AAI 2024 Careers in Immunology Fellowship to C.C. and R.V.

## Author contributions

R.V. conceived the study. C.C. and R.V. designed, executed, and analyzed experiments and wrote the manuscript. S.E.L. conceived the SpyCage scaffolds. A.M.M. and S.E.L. generated and provided SpyCage, *Py*MSP1(19), and *Py*UIS4 proteins.

## Declaration of interests

We wish to disclose the following two intellectual property claims related to this protocol: Lindner, SE & Hafenstein, S. US Patent Application 16/494,502 “Versatile Display of Proteins” and Lindner, SE., Hafenstein, S., & Butler, N. PCT/US2020/033785 “Specific Selection of Immune Cells Using Versatile Displace Scaffolds.”
